# Antibiotic Resistance Profile of Common Bacteria Isolated from Blood Stream, Lower Respiratory Tract and Urinary Infections in Intensive Care Unit in Saudi Arabia: A Retrospective Study

**DOI:** 10.4314/ejhs.v31i6.19

**Published:** 2021-11

**Authors:** Ahmed M Kabrah, Saeed M Kabrah, Fayez S Bahwerth, Naof F Alredaini

**Affiliations:** 1 Laboratory Medicine Department, Faculty of Applied Medical Sciences, Umm Al-Qura University, Makkah, Kingdom of Saudi Arabia; 2 Molecular Genetics, King Faisal Hospital, Ministry of Health, Makkah - Kingdom of Saudi Arabia; 3 Faculty Member, King Abdulaziz University in Rabigh, Rabigh - Kingdom of Saudi Arabia

**Keywords:** Antibiotic Resistance, Central venous catheter, Saudi Arabia, Isolated microorganisms, Intensive care unit

## Abstract

**Background:**

The rate of infections in the intensive care units (ICUs) is rising, mainly because of the increasing use of invasive procedures and specialized devices. This study aimed to identify the antibiotic resistance profile of common bacteria isolated from lower respiratory tract infections (LRTIs), bloodstream infections (BSIs), and urinary infections (UTIs) in ICUs in Saudi Arabia.

**Methods:**

In the current retrospective study, the isolates and antibiotic resistance were collected from the Electronic Medical Record (EMR) for respiratory, blood, and urine samples. The study sample compromised 96 patients admitted to the ICU at least for 48 hours and have a central venous catheter (CVC) between November 1, 2020, and January 31, 2021.

**Results:**

66 (68.8%) of the study sample were males, and 30 (31.2%) were females. LRTIs were the most common isolates (51 samples), followed by BSIs (28 samples) and UTIs (17 samples). The isolated pathogens in this study were Klebsiella pneumoniae (K. pneumoniae) (59.4%), Coagulase-negative staphylococci (CoNS) (11.5%), Escherichia coli (E. coli) (8.4%), Acinetobacter baumannii (A. baumannii) (7.3%), and Staphylococcus aureus (S. aureus) (6.2%). BSI were frequently caused by CoNS (35.7%) and K. pneumoniae (35.7%), while Methicillin-resistant Staphylococcus aureus (MRSA) represented 10.7% of BSI. Vancomycin, Synercid, and Teicoplanin were the commonly used antibiotics and showed 100% sensitivity among S. aureus, including MRSA, while almost 100% resistance was observed for penicillin and oxacillin against the same organisms. The maximum resistance was observed with aztreonam (96.4%), ampicillin (87.3%), followed by co-amoxiclav (83.9%), cotrimoxazole (79.5%) and cephalosporin group antibiotics.

**Conclusions:**

Strict adherence to infection prevention practices and wise use of antibiotics are recommended to slow the spread of antimicrobial resistance (AMR).

## Introduction

Intensive care units (ICUs) are the most life-saving facilities in hospitals, with highly specialized clinicians and nurses caring for severely ill patients. Most conditions treated in ICU are life-threatening, severe injuries and illnesses that necessitate regular monitoring and support from specialist technology and pharmaceuticals to ensure normal body processes (1, 2).

ICUs' infection rates are growing, owing to the increased use of invasive procedures and specialized gadgets (3). Antibiotic abuse and misuse, partly due to inaccurate diagnosis, and illogical antibiotic market combinations, are some of the factors that lead to the widespread drug resistance among hospital-acquired pathogens. The reasons include irregular consumption due to either a faulty prescription or poor compliance (4, 5).

Other risk factors, such as an extended hospital stay, appear to predispose patients to antibiotic-resistant bacteria infection (6). The occurrence of antibiotic-resistant bacterial infections aids the spread of antibiotic resistance due to inadequate infection control procedures and the failure to detect the presence of antibiotic resistance (7, 8).

Antibiotic resistance can be reduced by following antimicrobial stewardship program standards when using antibiotics [9]. *S. aureus, Enterobacteriaceae*, and *Pseudomonas species* are the most commonly reported pathogens in ICUs. *Enterococci, Candida, CoNS*, and *A. baumannii species* are bacteria (10, 11).

ICU-acquired infections have been linked to increased morbidity, mortality, and growing healthcare expenditures (12, 13). Antibiotic-resistant infections have been increasingly emerging and spreading in ICUs around the world. The overall infection rate is 23.7 infections per 1000 days in the ICU. ICU nosocomial infections account for around 20% to 25% of all nosocomial infections (14, 15).

Infection with *Gram-negative bacilli (GNB)* that produce *extended-spectrum beta-lactamase (ESBL)* and *carbapenemase* and an alarming number of multidrug-resistant isolates is a severe healthcare concern for ICU patients. To decrease the spread of AMR, firm adherence to infection prevention methods and prudent antibiotic use is recommended (16).

A high frequency of *multidrug-resistant ESBL* pathogens among pediatric patients was discovered in the International Friendship Children's Hospital, Kathmandu, Nepal. Stopping the spread of AMR and *ESBL* requires treatment based on their routine identification and susceptibility to certain antibiotics (17–19).

In Saudi Arabia, the *GNB* shows a significant increase in becoming carbapenem-resistant GNB, which is significantly higher than the other part of the globe. In addition, an increasing prevalence of *ESBL* has been found in Saudi Arabia. It has been further specified that *K. pneumoniae* shows 65% *ESBL*, and *E. coli* offers 29% *ESBL* rates. It is causing an increase in Saudi Arabia's mortality rate from 11% to 40% through multiple reported mortality outbreaks in uncontrollable infection due to AMR pathogen infections (20). The rate of *Carbapenem-resistant A. baumannii* (CRAB) has increased rapidly in the last five years (21). According to the recent surveillance conducted on Gram-positive cocci in Saudi Arabia, 32% of *S. aureus* has become *MRSA* entirely, 33% of *Streptococcus pneumoniae* (*S. pneumoniae*) have become resistant to penicillin G, and another 26% have become utterly resistant to erythromycin (20).

Due to the role of hospitals in delivering health care, evolving emerging infectious diseases, growing antibiotic resistance, extending the length of stay for hospital admissions, particularly in the ICUs and lack of accurate information and statistics on AMR, therefore, this article aimed to determine the antibiotic resistance patterns of isolated microorganisms in ICU patients in the King Faisal Hospital at Saudi Arabia.

## Methods

**Study design and data collection procedure**: In this retrospective study, the isolates and antibiotic resistance were collected for respiratory, blood, and urine samples. Data were collected from patients admitted to the ICU for 48 hours and have a CVC in the King Faisal Hospital between November 1, 2020. January 31, 2021, a standard data collection sheet was gathered from the Electronic Medical Record (EMR). Demographic and clinical data regarding the gender of patients and samples source were also collected. Blood drawing procedures have been done by hospital nursing, which follows the Ministry of Health regulation that includes cleaning the skin with 2% Chlorhexidine with 70% isopropyl alcohol applicator for 30 seconds using back and forth scrubbing technique. Besides, two sets of blood cultures were drawn from patients to roll out the contamination. For confirmation about the isolated bacteria as *CoNS*, the samples were sent to a reference lab for molecular identification.

**Sample size and sampling**: The current study comprised all hospitalized patients to the King Faisal Hospital's ICU for at least 48 hours and with CVC between November 1, 2020, and January 31, 2021. Accordingly, the study participants were selected using the census sampling method as all eligible patients found in the Electronic Medical Record (EMR) (96 patients) were included in this study.

**Illegibility criteria:** Patients only admitted between November 1, 2020, and January 31, 2021, to the ICU at the King Faisal Hospital for at least 48 hours and with CVC were qualified for the study to eliminate the community-acquired infection. Patients who had positive culture reports were only included in the study.

**Isolation and identification of pathogens**: King Faisal Hospital Microbiology laboratory proceed with the received samples from the ICU according to the standard operating procedures for bacterial isolation and identification. Blood culture bottles were incubated in Biomerieux BACT/ALERT. After the machine gave the growth signal, the samples were cultured in Blood, MacConkey and chocolate agar. Urine samples were cultured in Blood, MacConkey or CLED agar. Lower respiratory tract samples were cultured in Blood, MacConkey and chocolate agar. They have then incubated aerobically at 37°C for 24 to 48 hours except for the blood culture, which was incubated for 5 to 7 days. Preliminary identification of some isolates was performed based on the colonial morphology, Gram stain, and routine rapid biochemical tests such as catalase, indole, and oxidase tests. Bacterial identification was performed according to the protocols of the hospital where the strain originated: Gramnegative strains were identified by Pos Breakpoint Combo Panel Type 50 in MicroScan (Beckman Coulter Inc., CA, United States) and Gram-positive strains by Pos Breakpoint Combo Panel Type 28 in MicroScan (Beckman Coulter Inc., CA, United States).

**Antimicrobial susceptibility testing**: The susceptibility test of *Gram-Positive bacteria* was carried out according to the Clinical and Laboratory Standards Institute (CLSI). Identified strains were tested in vitro against several classes of antimicrobial drugs using the MicroScan automated microbiology system (Pos Breakpoint Combo 28 Panel). The following antimicrobial agents were examined: Amikacin (30 µg/ml), Amox/K clav (0.5 µg/ml), Azithromycin (30 µg/ml), Ampicillin (4 µg/ml), Aztreonam (16 µg/ml), Ciprofloxacin (2 µg/ml), Cefoxitin Screen (4 µg/ml), Clindamycin (2 µg/ml), Inducible Clindamycin (8 µg/ml), Daptomycin (4 µg/ml), Erythromycin (4 µg/ml), Fosfomycin (32 µg/ml), Fusidic Acid (16 µg/ml), Gentamicin (8 µg/ml), lmipenem (8 µg/ml), Levofloxacin (4 µg/ml), Linezolid (4 µg/ml), Moxifloxacin (1 µg/ml), Mupirocin (8 µg/ml), Oxacillin (2 µg/ml), Penicillin (8 µg/ml), Rifampin (2 µg/ml), Synercid (2 µg/ml), Teicoplanin (16 µg/ml), Trimeth/Sulfa (0.05 µg/ml), and Vancomycin (16 µg/ml). Quality control and maintenance were achieved according to the manufacturer's guidelines.

The susceptibility test of Gram-Negative bacteria was carried out according to the recommendations of the Clinical and Laboratory Standards Institute (CLSI). Identified strains were tested in vitro against several classes of antimicrobial drugs using the MicroScan automated microbiology system (Pos Breakpoint Combo 50 Panel). The following antimicrobial agents were examined: Amikacin (30 µg/ml), Amox/K clav (2 µg/ml), Amp/Sulbactam (2 µg/ml), Ampicillin (16 µg/ml), Aztreonam (16 µg/ml), Cefazolin (4 µg/ml), Cefepime (16 µg/ml), Cefotaxime (16 µg/ml), cefoxitin (16 µg/ml), Ceftazidime (16 µg/ml), Ciprofloxacin (2 µg/ml), Cefuroxime (16 µg/ml), Colistin (4 µg/ml), Ertapenem (1 µg/ml), Gentamicin (8 µg/ml), lmipenem (8 µg/ml), Levofloxacin (4 µg/ml), Meropenem (8 µg/ml), Moxifloxacin (1 µg/ml), Nitrofurantoin (64 µg/ml), Norfloxacin (8 µg/ml), Pip/tazo (16 µg/ml), Tigecycline (2 µg/ml), Tobramycin (8 µg/ml), and Trimeth/Sulfa (0.05 µg/ml).

**Ethical considerations**: Ethical approval for this study was obtained from the Research and Ethical Committee of the College of Medicine at Umm Al Qura University (HAPO-02-K-012-2021-08-713). Also, permission was obtained from the King Faisal Hospital to access the Electronic Medical Record (EMR) to get the required data using a standard data collection sheet.

**Statistical analysis**

The Statistical Package for Social Science (SPSS) version 24 (IBM Corp, Armonk, NY, USA) was used for data analysis, including descriptive statistics of frequencies and percentages.

## Results

A total of 96 patients were included in this study. All the clinical specimens were from patients admitted to the ICU for at least 48 hours. Among the samples, 66 (68.8%) were males, and 30 (31.2%) were females. Patients who had positive culture reports were only included in the study.

To determine the distribution of infection, the major sites of infection were examined. All the patients had fallen into either one of the three major sites of infection, such as LRTIs, BSIs, or UTIs. LRTIs were the most common (51 samples), followed by bloodstream infection (28 samples) and urinary tract infection (17 samples).

BSIs were frequently caused by *CoNS* (35.7%) and *K. pneumoniae* (35.7%), while *MRSA* represented 10.7% of bloodstream infections. Nearly two-thirds of the reported isolates (64.7%) from the UTIs were *K. pneumoniae*, while *E. coli* was isolated in 23.6% (Table 1).

**Table 1:** Prevalence of organisms in different sources

Sample	Coagulase- negative *staphylococci*	*Klebsiella* *oxytica*	ESBL-*E coli*	*E-Coli*	*Enterobacter* *aerogenes*	*Acinetobacter* *baumanni*	*Staphylococcus* *aureus*	*Serratia* *marcescens*	*Pseudomonas*	*Morganella* *morganii*	MRSA	ESBL-*Klebsiella*	*Klebsiella* *pneumoniae*	Total
**Blood**	10 (35.7)	0 (0)	0 (0)	2 (7.1)	0 (0)	0 (0)	2 (7.1)	0 (0)	0 (0)	1 (3.6)	3 (10.7)	1 (3.6)	9 (32.1)	28 (100)
**Respiratory**	1 (2)	2 (4)	2 (3.9)	0 (0)	0 (0)	7 (13.7)	1 (2)	0 (0)	2 (3.9)	0 (0)	0 (0)	2 (3.9)	34 (66.7)	51 (100)
**Urine**	0 (0)	0 (0)	1 (5.9)	3 (17.6)	1 (5.9)	0 (0)	0 (0)	1 (5.9)	0 (0)	0 (0)	0 (0)	1 (5.9)	10 (58.8)	17 (100)
**Total**	11 (11.5)	2 (2.1)	3 (3.1)	5 (5.2)	1 (1)	7 (7.3)	3 (3.1)	1 (1)	2 (2.1)	1 (1)	3 (3.1)	4 (4.2)	53 (55.2)	96 (100)

*K. pneumoniae* were the most frequently infecting organisms representing more than half of the infection. A total of 57 (59.4%) patients were infected with *K. pneumoniae*, including five specimens representing the *ESBL* group. The most frequent site of infection for *K. pneumoniae* was the respiratory tract, followed by urinary and bloodstream infection.

Next to *K. pneumoniae*, the percentages of the most commonly isolated organisms were as follows: *CoNS* (11.5%), *E. coli* (8.4%), *A. baumannii* (7.3%) and *S. aureus* (6.2%). All these five organisms were positive in 92.8% of specimens examined. Moreover, it is essential to note that half of the *S. aureus* cultures have shown *MRSA*, while *ESBL* positivity was observed among 37.5% of the *E. coli* culture (Table 2).

**Table 2:** Frequency and percentage of type of microorganisms in the study

Organism	Frequency	Percentage
***Coagulase-negative staphylococci***	11	11.5
***Klebsiella oxytica***	1	1
***ESBL-Klebsiella oxytoca***	1	1
***ESBL-E coli***	3	3.1
***E-Coli***	5	5.2
***Enterobacter aerogenes***	1	1
***Acinetobacter baumannii***	7	7.3
***Staphylococcus aureus***	3	3.1
***Serratia marcescens***	1	1
***Pseudomonas***	2	2.1
***Morganella morganii***	1	1
***MRSA***	3	3.1
***ESBL-Klebsiella***	4	4.2
***Klebsiella pneumoniae***	53	55.2
**Total**	96	100

Antibiotic susceptibility test was applied according to the type of isolates in the specimens. Among the commonly used antibiotics, vancomycin, Synercid, and teicoplanin showed 100% sensitivity among *S. aureus*, including *MRSA*, while almost 100% resistance was observed for penicillin and oxacillin against the same organisms. However, amikacin, piperacillin-tazobactam and gentamycin did not show much sensitivity against *Enterobacteriaceae*.

Among the other antibiotics, maximum resistance was observed with aztreonam (96.4%), ampicillin (87.3%), followed by co-amoxiclav (83.9%), cotrimoxazole (79.5%) and cephalosporin group antibiotics. Meropenem had the least prevalence of antibiotic resistance (7%), although it is not widely tested in gramnegative organisms in this study (Table 3).

**Table 3:** Overall percentage of antimicrobial resistance identified in this study

Antimicrobial	%	Antimicrobial	%	Antimicrobial	%	Antimicrobial	%
Amikacin	73.4	Ceftazidime	81.4	Piperacillin/tazobactam	77.4	Synercid	0
Co-amoxiclav	83.9	Cefuroxime	83.6	Tobramycin	76.5	Teicoplanin	0
Azithromycin	68.4	Gentamycin	75.3	Cotrimoxazole	79.5	Vancomycin	0
Ampicillin	87.3	Levofloxacin	52.1	Penicillin	100	Tobramycin	76.8
Aztreonam	96.4	Meropenem	7	Oxacillin	88.2	Nitrofurantoin	70.5
Cefotaxime	88.6	Imipenem	66.7	Rifampin	23.5	Norfloxacin	64.7

Table 4 shows the percentages of AMR identified in this study for the commonly used antibiotics in clinical practice.

**Table 4:** Antibiotic resistance profile of gram-positive cocci, data were presented and frequency and percentage (%)

Organism	Number of Isolates	Penicillin	Oxacillin	Rifampin	Mupirocin	Vancomycin	Teicoplanin	Cinercid
***Coagulase-negative staphylococci***	11	11 (100)	10 (90.9)	4 (36.4)	2 (14.2)	0	0	0
***Staphylococcus aureus***	3	3 (100)	2 (66.6)	0	0	0	0	0

## Discussion

This is the most recent report of the antibiotic resistance profile of common bacteria isolated from LRTIs, BSIs, and UTIs in the ICU in Saudi Arabia. This study used Modified Disk Diffusion Method (MDDM) by Mueller-Hinton agar medium to isolate antibiotic resistance. Patients who had positive culture reports were only included in the study.

Male patients made up more than half of the participants in this study. The possible explanation might be that the King Faisal Hospital ICU is predominantly populated with infected male patients than female patients. Furthermore, LRTIs were the most common isolates (51 samples), followed by BSIs isolates (28 samples) and UTIs isolates (17 samples). This result is consistent with earlier research in Riyadh capital and Southwest Saudi Arabia, where most bacterial isolates found in ICU patients came from the respiratory tract (22, 23). Our findings are consistent with the study from Nepal, which showed bacterial growth in specimens from patients suspected of hospitalacquired pneumonia (16%, 79/491), BSIs (5.7%, 28/491), and UTIs (3.9%, 19/491) (24). Surveillance studies in ICUs also showed that most bacterial isolates were from the respiratory tract (25, 26).

The isolated pathogens in this study were *K. pneumoniae* (59.4%), *CoNS* (11.5%), *E. coli* (8.4%), *A. baumannii* (7.3%), and *S. aureus* (6.2%). Bloodstream infections were frequently caused by *CoNS* (35.7%) and *K. pneumoniae* (35.7%), while *MRSA* represented 10.7% of bloodstream infections. Nearly two-thirds of the reported isolates (64.7%) from the urinary tract were *K. pneumoniae*, while *E. coli* was isolated in 23.6%. The most frequent site of infection for *K. pneumoniae* was the respiratory tract, followed by UTIS and BSIs.

These findings could be compared with those reported in a retrospective study at a tertiary care hospital in Southwest Saudi Arabia. *Acinetobacter spp*. (27.2%), *P. aeruginosa* (23.8%) and *K. pneumoniae* were the most often isolated pathogens (18.6%) (23). Another retrospected cohort study conducted at Riyadh Military Hospital revealed that *A. baumannii* accounted for 40.9% of all ICU isolates, followed by *K. pneumoniae* (19.4%) and *Pseudomonas aeruginosa* (16.3%) (22). Consistently, such *Gram-negative bacteria (GNB)* isolates were the most frequent pathogens in 6 years of epidemiologic surveillance in Saudi Arabia for ventilator-associated pneumonia at a tertiary-care ICU (27). The predominance of GNB in ICUs was also reported in surveillance programs conducted in Europe and the USA (28, 29). The spread of these etiological agents might be linked to contaminated respiratory equipment and transmission via the hands of healthcare workers in ICU settings. Therefore, the transmission of GNB in ICUs could be prevented by reinforcing appropriate control measures coupled with examining the rate of bacterial contamination of the hands of healthcare workers and the ICU environment (30).

In the present study, among the commonly used antibiotics, vancomycin, Synercid, and teicoplanin showed 100% sensitivity for *S. aureus*, including *MRSA*. In comparison, almost 100% resistance was observed for penicillin and oxacillin against the same organisms. Among the other antibiotics, maximum resistance was observed with aztreonam (96.4%), ampicillin (87.3%), followed by co-amoxiclav (83.9%), cotrimoxazole (79.5%) and cephalosporin group antibiotics. In this study, Meropenem had the least prevalence of antibiotic resistance (7%), although it is not widely tested in gram-negative organisms.

The presence of diverse resistant pathogens is an emerging clinical problem in ICUs (26). In comparing the isolates from the 3 ICUs, we found that *Acinetobacter spp*. are the most frequent isolates from the adult ICU. In contrast, reports from African countries and India found that *Staphylococcus spp*. were the predominant isolates from patients in the ICU (23, 31–33). Horizontal transmission from infected visitors or healthcare staff to the juvenile population in the ICU (31).

The multi-drug-resistant (MDR) rates are questionable, which makes ICU infections caused by GNB difficult to treat in our setting. Non-compliance with hand hygiene among healthcare staff transmitted MDR strains from patient to patient in ICUs, according to research conducted at Aseer Central Hospital in Abha in the country's southern area (36). In addition, MDR strains have emerged in ICUs (34) due to cross infections among inpatients, ICU procedures, and patients with chronic diseases (16, 22, 26). Moreover, prolonged hospital ICU stays combined with unnecessary antibiotic treatment may hasten the development of MDR infections.

Available literature reports that antibiotic use has a significant relationship with AMR (35–37). This resistance might be developed as a result of misuse of antibiotics. Several studies have reported that the antimicrobial prescribing pattern of physicians is a determinant of increasing resistance to antibiotics (38, 39).

As a result, the common risk factors that might be linked to rising MDR trends across ICU infections should be determined in our institution. In addition, it is critical to set a precise antibiotic schedule and analyze the resistance pattern in the hospital regularly to prevent the future spread of multidrug resistance and boost antibiotic effectiveness.

This is the most recent study to investigate the antibiotic resistance profile of common bacteria isolated from bloodstream infection (BSI), lower respiratory tract (LRTI) and urinary infection (UTI) in ICUs in Saudi Arabia. The findings of this study will be a valuable resource for policymakers and clinicians interested in learning more about the antibiotic resistance profile of common bacteria isolated in Saudi Arabian ICUs, which will help them determine the best treatment, antibiotic use, and infection control strategies.

While our findings indicated that more than half of the patients were infected with *K. pneumoniae*, including five specimens representing the *ESBL* group, the maximum antibiotic resistance was observed with aztreonam, ampicillin, co-amoxiclav, cotrimoxazole, and cephalosporin group antibiotics. Surveillance aimed at the regional and national levels for a complete picture of Suadi Arabia may have bolstered the findings. In addition, Patients who had positive culture reports were only included in the study; this blocks the chance of showing the real burden of BSI, LRTI and UTI among eligible patients admitted in the ICU during the study period.

It is concluded that in our ICU, respiratory infections were more frequently than bloodstream and urinary tract infections. The isolated pathogens in this study were *K. pneumoniae* (59.4%), *CoNS* (11.5%), *E. coli* (8.4%), *A. baumannii* (7.3%), and *S. aureus* (6.2%). Vancomycin, Synercid, and Teicoplanin were the commonly used antibiotics and showed 100% sensitivity among *S. aureus*, including *MRSA*, while almost 100% resistance was observed for penicillin and oxacillin against the same organisms. The maximum resistance was observed with aztreonam, ampicillin, coamoxiclav, cotrimoxazole, and cephalosporin group antibiotics. Finally, the proper administration of antibiotics to ICU patients based on the standard antimicrobial susceptibility tests is suggested to reduce multidrug resistance.
